# Supplementary material for: Conditional chemoconnectomics (cCCTomics) as a strategy for efficient and conditional targeting of chemical transmission
Source: eLife. 2024 Apr 30;12:RP91927. doi: 10.7554/eLife.91927 (PMC11060718; doi:10.7554/eLife.91927)
Supplement: Figure 3—source data 3. [file elife-91927-fig3-data3.zip › Figure 3 source data3 part 1.zip!/Figure 3-Source data 3-part1/1082-9-CRISPResso_Report_zod2ewbs.zip!/CRISPResso_on_1082_10/4b.Insertion_deletion_substitution_locations.pdf]

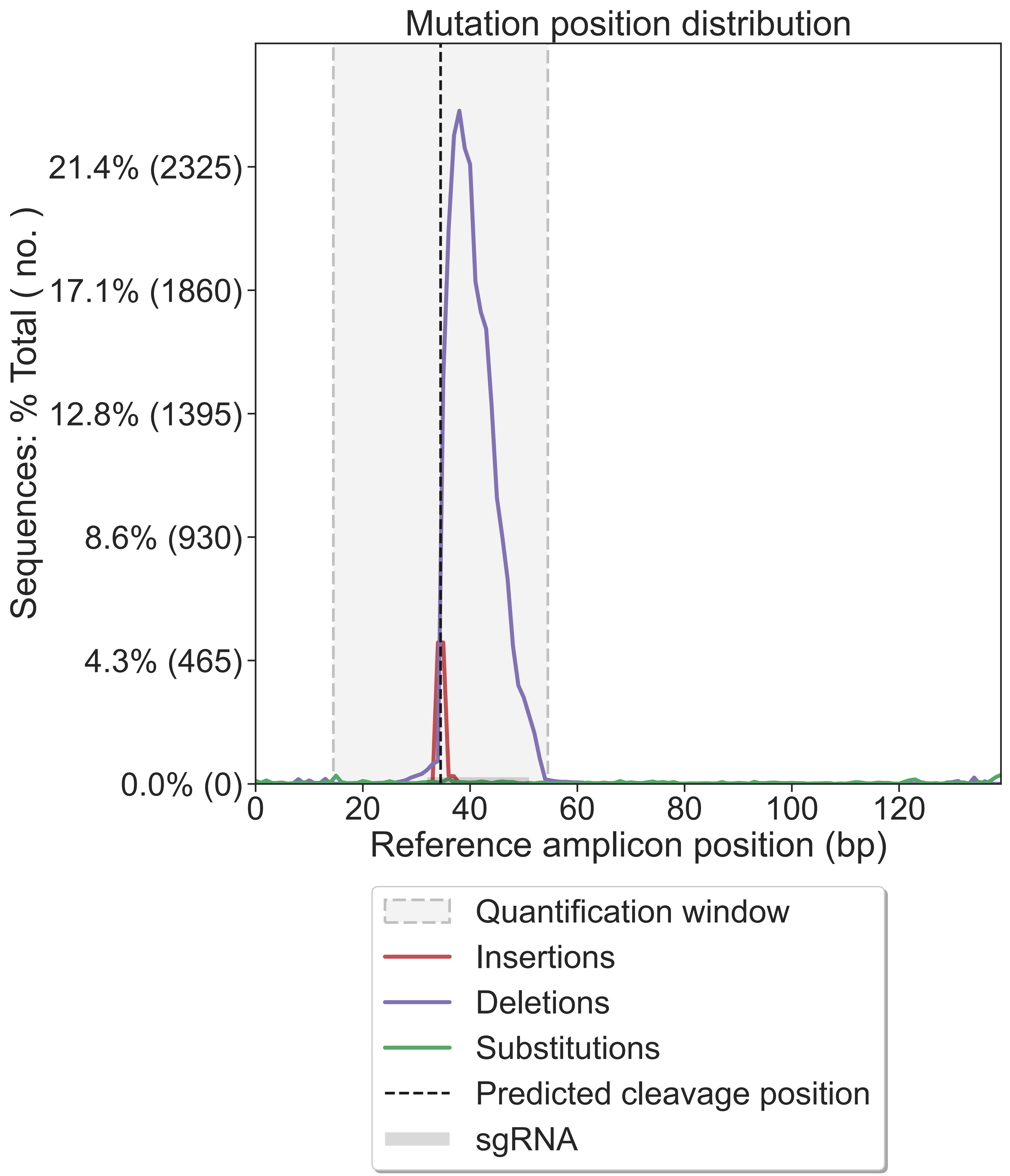

Mutation position distribution
21.4% (2325)
17.1% (1860)
12.8% (1395)
8.6% (930)
4.3% (465)
0.0% (0)
Sequences: % Total (no.)
0
20
40
60
80
100
120
Reference amplicon position (bp)
Quantification window
Insertions
Deletions
Substitutions
Predicted cleavage position
sgRNA
